# The Relation of Medicine to Civilisation1President’s anniversary address, delivered before the Medical Society of the State of New York, February 1, 1899.

**Published:** 1899-07

**Authors:** John O. Roe

**Affiliations:** Rochester, N. Y.


### THE RELATION OF MEDICINE TO CIVILISATION.[1]

By JOHN O. ROE, M. D., Rochester, N. Y.

IT IS the privilege of the present generation to witness the most brilliant achievements in science, literature and the arts, that the world has ever known. This epoch may be regarded as the consummation of the ages, the result of all previous endeavors, and the refined product of all previous thought.

1. President's anniversary address, delivered before the Medical Society of the State of New York, February 1, 1899.

Of all the different factors that have contributed to this result, science may be regarded as the most important, the greatest civiliser of them all, whose progress may be likened to the motion of a falling body which is increasingly accelerated by its accumulated momentum.

The influence of the marvelous discoveries in science during the past quarter of a century in advancing civilisation is shown in the increased general enlightenment of the people; in the stimulation of original investigation; in the liberalisation of public sentiment; and in freeing the mind from the dogmas of the past. This general advance in scientific knowledge, however, has resulted not from progess in any one department, but from the simultaneous discoveries in each, from the consensus of the sciences, as Spencer terms it, each contributing to the advancement of all the others. But no department has contributed so much to civilisation in the prolongation of life and the general happiness of mankind, as that of medicine.

I would, therefore, ask your attention to a few thoughts on the relation of medicine to civilisation, noting first, the influence of civilisation on medicine, and second, the influence of medicine on civilisation.

### THE INFLUENCE OF CIVILISATION ON MEDICINE.

Every department of science, from the earliest times, has been dominated or influenced by the spirit of the times in which it existed. A cursory glance over the world's history shows that the intellectual progress of man has passed through three stages: mythological, metaphysical and scientific; each, however, merging slowly and almost imperceptibly into the other.

These three phases of thought have existed together in all ages, but each stage of intellectual development is marked by the predominance of one of these over the other two. Thus, with the vast majority of the early Egyptians, Greeks and Romans, mythology dominated all their actions in every form of life; and all the phenomena of nature were regarded by them as the result of the direct agency of the gods. Nothing was ever undertaken without first consulting the oracle of the god who was supposed to preside over this or that particular class of things or events.

A most ancient and revered law declares that "Sacred things can be revealed only to the elect, and ought not to be confided to the profane until they are initiated into the mysteries of the science;" and these elect were initiated only upon oath that they would keep the mystery. Thus the healing art was made hereditary and kept within the family, as is illustrated by the *Asclepiades*.

In early Egypt the only medical advice obtainable was from the passers-by, the patient being placed by the roadside; but later it was the law for them to be carried to the temple and to the priest, and, if cured, the symptoms and treatment were recorded for future use.

The scepticism that arose about the fourth century before the Christian era tended to do away with mythology, and opened the way for the philosophic and scientific stages that followed. For it was out of the desire and efforts to explain the various phenomena of mind and matter, that the different schools of philosophy grew up. The earlier thinkers who surpassed the common people in thought called themselves sophists (wise men); Socrates, however, not so pretentious, called himself a philosopher (lover of wisdom), and constructed many theories to account for the phenomena of which he was cognisant.

The different systems of philosophy, however, when compared, were found to be essentially opposite in their teachings so that doubt was cast upon all alike, and the thoughtful minds of the times turned in search of something that could be relied upon, which led to the growth of what may be called the scientific spirit. Now it may be said that

> Philosophy, that reached the heavens before,
> Shrinks to her hidden cause, and is no more.

Socrates, who held that all philosophy was vain which was not founded on fact, was followed by Plato, Aristotle, and others, who made investigations in many fields of knowledge, which, especially in the case of Aristotle, resulted in useful discoveries in zoölogy, anatomy and botany, departments of knowledge that had not before reached the dignity of positive science. Then followed the establishment of many schools and libraries.

Hippocrates was the first to infuse into the medical philosophy of his time the spirit of investigation, which, however limited in extent it may have been, was, nevertheless, the motive force and inspiration of the thinking minds of his time, planting the germ of the new science.

Stimulated by this spirit, Alexander the Great founded the Alexandrian library, which, under the direction of his esteemed tutor Aristotle, made itself felt in the works of Erasistratus and Herophilus in medicine, and of many others in the various departments of learning. Even after the final destruction of this library and university in 640 A. D., its influence was still alive in the planting of the great schools at Granada, Cordova and Toledo, in Spain, and at Salerno and other places in Italy, which preserved the science of medicine through the dark ages.

This three-fold influence of mythology, philosophy and science is found repeated in the history of the middle ages, the renaissance, and the last three centuries, respectively. For instance, all the persecutions of the dark ages were the result of this mythological spirit mingled with superstition, which sought to destroy the sciences in their infancy, as illustrated by the persecution of Galileo for his astronomical observations, and Roger Bacon for his physical and medical pursuits.

The renaissance was a quickening of the intellectual life, but with this quickening the mind was turning to the asthetic arts and the speculative sciences, to the neglect of the empirical sciences, and medicine consequently made little or no progress until Francis Bacon instituted the inductive method of study, which was the beginning of progress in the natural sciences, and has made the seventeenth, eighteenth and nineteenth centuries the really scientific period of the world's history.

In accordance with the spirit of the times, many laws were enacted retarding the growth and development of medicine. For many centuries all effort at dissecting was regarded as sacrilegious and was forbidden, owing to false conceptions and prejudices regarding the sacredness of the body. And these restrictions continued during the middle ages and the time of the crusades, and in 1317 Pope John XXII. issued a bull against alchemy, which was really executed against ortho-sciences and pseudo-sciences indiscriminately, and all experimental sciences were forbidden. In 1404 Pope Innocent VIII. issued a bull which let the Inquisition loose on Germany, with Sprenger in the lead, to destroy men and women by the tens of thousands for sorcery and magic; but every phenomenon was magic that was not understood; so that chemistry and physics and all experimental sciences, medicine among them, were very greatly retarded. There were many other similar enactments, and Luther himself was a profound believer in witches; the Reformation, even, giving no place to science.

Besides these ecclesiastical decrees, there were some civil decrees against the sciences. In 1380 Charles V., of France, forbade the possession of furnaces and apparatus necessary for the study of chemistry. In 1404 Henry IV., of England, issued a similar decree, and in 1418 Italy and the Republic of Venice followed these examples, while physicians were very generally regarded with suspicion and often massacred.

From considering the general influence of the spirit of the times in retarding the progress of medicine, we may now briefly consider what assistance medicine has derived from civilisation and especially from the other sciences.

Herbert Spencer says: "We find that to make a single good observation in the purest of the natural sciences requires the combined aid of half a dozen other sciences." It is this increasingly comprehensive consensus of the sciences that marks their development, and makes it impossible to draw a distinct line of demarcation between them, or to tell where one begins and the other ends. Thus Faraday's discovery of the laws of magneto-electric induction and polarity, led him at once to ask what is the relation of this polarity to chemical combination, and this launched him into long tasks of chemical research.

From physics alone medicine has appropriated very much to its use. Under the subject of light, we notice that Euclid's discovery (260 B.C.) that light travels in straight lines, finally resulted after many centuries in Alhazen's discovery (1030 A.D.) of the principle of refraction, reflection and magnifying glasses, and that the nature of sight was a physiological phenomenon. The experiments and discoveries of Roger Bacon and Vitellio (1240) of the nature of light and the causes of the rainbow, etc., led to Baptiste Porta's invention of the camera obscura (1560), from the application of which comes the science of photography, which has a very practical application to the medicine of today. From Alhazen's magnifying glasses came Jansen's microscope (discovered 1590), which was applied by Malpighi, in 1661, to the study of the cells of the lungs, resulting in his discovery of the Malpighian layer, and which led to the study of cells and glands throughout the body. By studying under the microscope the circulation in the mesentery of a frog, he discovered the nature of the circulation and the contraction of the blood corpuscles, as they were then called, on passing the walls of the cells. What would not Harvey have given to see this!

Microscopy has introduced us to a new world, revealing the minute structures of the body and the organisms that play so great a part in the plan of nature in health and disease, and, through the labors of the immortal Pasteur, has given to science a new department—bacteriology.

Under optics we may also mention Helmholtz's discovery (1851) of the principles of the ophthalmoscope, thus giving rise to ophthalmoscopy and ophthalmology. The laryngoscope, invented by Garci- and perfected by Czermak, has given precision and accuracy to the diagnosis of diseases of the larynx, nose and throat.

Newton's discovery of the nature of the solar spectrum (1670) was followed by the analysis of the spectrum by Wallaston and Frauenhofer (1802 and 1804). This was immediately applied by Sir William Herschel, not only to discoveries in the composition of the heavenly bodies, but to the discovery of heat rays, and the spectroscope, so extensively used today, and first applied by him to chemical analysis (1822); and it was by pursuing this same line of investigation that Röntgen was led to the discovery of the so-called *x*-rays, which has marked a distinct epoch in surgery.

Acoustics have contributed much to medical and surgical diagnosis.

From the earliest time sound was regarded by philosophers as a kind of entity, until Newton, in 1687, pointed out that it was caused by the vibrations of the particles of an elastic medium, varied according to the density of the medium, and this fact was first put to practical use in medical diagnosis by Auenbrugger in his discovery of percussion. This discovery, together with the application of the same principle to auscultation and the invention of the stethoscope by Laennec, revolutionised the diagnosis and treatment of internal diseases, and especially those of the chest. The various modifications which the stethoscope has undergone have finally culminated in the phonendoscope of Bianchi.

This principle of sound conduction is utilised in various methods of diagnosis in surgery for the detection of liquid and solid accumulations, for the location of bullets and the detection of foreign bodies in all the different cavities of the body; and it is also of special service in the diagnosis and treatment of aural affections.

The application of the principles of hydrostatics, in determining specific gravity in practically the same manner as that employed by Archimedes (250 B. C.), is at the present time one of the principal methods of determining the relative weights and measures of both solids and liquids, especially in the laboratory, where perfect accuracy is required, and is daily used in analysis for the detection of adulteration of foods.

The important principle of hydraulics (discovered by Pascal in 1563), that water will escape from an orifice at the bottom of a tank with the same velocity as if falling from the top, is applied to medicine by the invention of the fountain syringe.

From pneumatics, Guericke's air-pump, as improved by Boyle in 1642, is applied in the form of the exhaust stomach-pump, force-pump and ordinary syringe. After the invention of the mercurial barometer by Torricelli (1644), the thermometer (said to have been invented by Galileo long before, but in a very crude form) was improved and became the instrument of precision now so universally used in medicine, affording accurate measurement of temperature and data of the greatest value in the diagnosis, prognosis and treatment of disease.

The invention of the barometer led also to the study of atmospheric pressure and conditions affecting it, and, after the addition of Boyle's law of the compressibility of gases, came the study of climatology. Associated with this came also the experiments of Mayow on respiration, which led to the discovery of auscultation and the stethoscope by Laennec.

Archimedes's screw and lever have actually moved the world. All forceps, pliers and scissors are the application of the lever, and the screw is essential to innumerable surgical instruments. The introduction of the balance, a simple lever, in chemical experiments in the eighteenth century, resulted in an exactness never before reached, and quantitative analysis of today owes its scientific value to its application. It was the application of quantitative analysis that overthrew the phlogistic theory and led Lavoisier to the discovery of oxygen.

Electricity has contributed its part to the advancement of modern medicine.

Gilbert (1540-1603), discovered frictional electricity, which led to the discovery of animal magnetism, and which in turn marked an epoch in medicine. In 1789 Galvani discovered animal electricity, as we remember, by his wife calling his attention to the twitching of the leg of the frog she was dressing for dinner, when the wire from an electric machine happened to be brought in contact with it. Volta, making experiments to disprove this fact, and to show this phenomenon to be chemical instead of animal, did not disprove galvanic electricity but discovered chemical or voltaic electricity.

From using voltaic electricity, Carlisle and Nicholson (1800) discovered chemical analysis of water by the electric current, which led Davy to apply this mode of analysis to all substances, giving an exactness not reached by Bermann with his substitution method. It was in following Davy's experiments, that Faraday discovered the intimate connection between electricity and chemical affinity, and invented the voltameter (1834).

Of the many contributions of chemistry to medicine, only a few facts need be mentioned here.

The most material general results of the study of the chemistry of gases (after the discovery of oxygen and the explosion of the phlogistic theory), were (1) that gases are to be numbered among the constituent elements of solids, which was applied with great advantage to physiology, and (2) that the compound is equal to the sum of all its constituent parts, which led to the methods of quantitative analysis, of so great moment in medical science of today.

Contemporary with Faraday, the chemist Wöhler (1838) discovered that urea, which is an organic substance in the bodies of all animals, could be made artificially, and this has resulted in the manufacture of the various organic synthetic compounds now so extensively employed for therapeutic purposes. Baron Liebig, of Darmstadt, traced the chemical changes that food undergoes in the body, showing which made fat, which muscle, etc., thus enabling us to supply the appropriate nutrition.

From the discoveries of bacteriology we have, through chemistry, a knowledge of how the poison produced by microbes affects the body, and of the means, as yet only in their infancy, for counteracting their effects and for guarding against them by inoculation and serum therapy.

From these discoveries it is also found that there is a special causative microbe in the common contagious and infectious diseases, such as typhoid and malarial fever, diphtheria, cholera and pulmonary consumption and also in all forms of surgical infection. Every discovery in bacteriology, therefore, contributes materially to the progress of medicine and surgery.

The advance of botany and the more accurate methods of study and classification of plants have influenced medicine not a little. The founding of the Natural History School of Medicine by De Candolle (1778-1841), of Genoa, and by Endlicher, of Vienna, has given additional aid to medicine, and from the impulse given by this school came the discovery of the cells of plants and of animals by Schleiden, Baumgarten and by Schwann respectively, from which has orginated the present teaching as to the histology and pathology of cell-life and growth.

Psychology and sociology have also contributed much in recent years for the relief of many mental and social conditions affecting society.

Psychology, reaching a high degree of scientific perfection of late years, has become of very great importance to the science of medicine in determining the relation of the mind to the body and throwing new light on the obscure processes of the brain and nerves.

It is known that among the Greeks the nervous excitement connected with the worship of their many gods (numbering, in Athens alone, 3,000 at the beginning of the Christian era), and also with the dissipation connected with their bacchanalian revels, linked with ignorance and superstitious dread of the unseen, go far toward accounting for the functional nervous diseases and insanity among them, which, in the age of Pericles, took the form of suicide among the women, many hundreds committing suicide in a year. Analogous to this were the epidemics of hysteria and insanity of the middle ages throughout Italy, Germany, Holland, and the Netherlands; at Cologne there were 500 afflicted at one time; at Metz there were 1,100 dancing in the streets, and at Strasburg still more, and from these centers the excitement became contagious and spread throughout the country and villages. I have no doubt that many of the monstrosities of the dark ages are to be accounted for on the grounds of insanity, mingled with debauchery and deviltry. The ecclesiastics of the time ascribed it to demonical possession, and the laws enacted for the punishment of persons so afflicted, together with the prohibition of scientific investigation, and the silencing of medical opinion on the subject, allowed all insane manifestations to go without scientific treatment for many centuries.

It is due to the scientific spirit of the nineteenth century, that we have learned to look for the causes of insanity in the diseased condition of the body, rather than in obscure mental disturbance and supernatural influences; that mental action is always and everywhere accompanied by a process of nervous change, and that this connection between mind and body, whether in health or disease, is uniform, the study of which connection must yield valuable results in the future.

From a sociological point of view, scientists are already looking at crime as a result of the conditions of society and natural degeneracy. This last falls especially in the domain of medical science, and the other is associated with it. The physical basis of criminality is a feature more and more recognised, and any reform, that would do effectual work in uplifting human society, must begin by bettering the conditions of *living*, in order that the conditions of *thinking* and *acting* may be improved. If trouble were taken, there is little doubt that many of the crimes of today could be traced to poverty, or disease, or both, for they are closely related. We recognise a class of hereditary criminals who have the natural impulse for crime; one man goes to the penitentiary or gallows, while another, whose mental derangement is no greater, but of a different kind, is treated for insanity. Will not civilisation make another step in the direction of treating crime in accordance with true science and truer justice than now obtains?

Already Chicago and Elmira have institutions for the correction of boy criminals. May not this be extended further? We still have work for John Howards in our prisons, and for Victor Hugos to write with master hand the story of the Jean Valjeans and Fantines of the present time.

We find that not only do sciences affect each other directly, but also indirectly. Even when there is not dependence there is analogy, which suggests likeness; and where a set of relations is discovered to exist in one of the sciences, at once research is made to find similar relations in all the sciences.

Since medical science is so composite, and from its very nature can, like a powerful magnet picking up iron filings chipped off from the other sciences, draw material from them all, and especially from those of which I have spoken, it is almost superfluous for me to emphasise the paramount importance of a thorough knowledge of all these different branches of science, in order to be able to utilise their discoveries and inventions in the prevention and treatment of disease. Moreover, from the fact that all the sciences are related and have always been seen to advance together, we have a suggestion of the necessity of a knowledge outside the limits of our own profession. The very principle of specialisation implies a knowledge of other things. We ought to know other sciences also, because of the common aim of them all—the acquisition of truth, the betterment of the conditions of humanity, and the advancement of civilisation.

### THE INFLUENCE OF MEDICINE ON CIVILISATION.

Having thus briefly noticed, on the one hand, what medicine has derived from civilisation, let us now turn to consider, on the other, what medicine has contributed to civilisation.

I think it will be seen that, in proportion to the investment, civilisation has derived from no other source so great dividends as it has from medicine—both for the life and health of humanity and the economic prosperity of the nation. Not only does science civilise, as was said in the beginning, but it is equally true that civilisation reacts on science, and thus the two are mutual helpers toward that which is better and beyond. In this connection we will note first the public benefits derived from medical science in the prevention of disease, and, second, in the cure of disease—that is, preventive and curative medicine.

The first most noticeable effect of the study of the nature and the conditions affecting the propagation and spread of disease, is seen in the modification and disappearance of the epidemics of the middle ages, that swept nations and continents before them.

The most notable illustrations are: The plague, which followed the destruction of Pompeii and Herculaneum by the eruption of Mt. Vesuvius—10,000 persons dying daily. The plague of Antoninus, 166 A. D., which depopulated many cities of the Roman Empire. The Justinian plague, from 542 to 549 A.D., which devastated all Europe and Asia. The world-wide pestilence of 744 A.D. which which carried off millions of people. The black death of 1348-49, from which, in Europe alone, Hecker estimates 25,000,000 perished. The London plague of 1665 from which 100,000 people perished. The sweating sickness in England, which followed the war of the Roses in 1485 was no less fatal, and the bubonic plague of the East caused millions of deaths in the eighteenth century.

These epidemics were due, not alone to the lack of knowledge respecting the nature of the diseases, but also to the system of crowding people closely together in walled cities, which, with the narrow streets, and lack of ventilation and sewerage, and contaminated water-supply, afforded a splendid breeding-ground for the germs of disease. After a time, however, attention was directed to the relation between these conditions and the spread of disease, particularly after the great London fire in 1666, which not only destroyed the buildings, but the filth as well. And when the city was rebuilt with wide streets and better constructed houses, it was observed that the public health was very greatly improved.

The effect of one disease in exterminating another is illustrated in the case of leprosy, which had prevailed in England to a great extent for thirteen centuries, and had subsided somewhat after the famine of 1315 and 1316 and the black death of 1349, but was completely exterminated by the plague of 1665, "which three together," says Dr. Ewart, "carried off more than one-half the population of the British Isles."

From the observation that the health of the community depended upon the purity of the water-supply came the knowledge that many of our infectious diseases were in that way communicated, as in the case of typhoid fever, cholera, and the like, and the discovery that the contagious principle was due to germs resulted in our present excellent system of sanitary regulations, guarding the public against all forms of infectious diseases. The belief that the causes of many diseases were disseminated through the atmosphere led to the study of the different forms of malarial diseases found to be associated with damp or marshy lands, stagnant waters, and damp dwellings. This led to the proper drainage of these regions and to domestic sanitation, and to the discovery of the plasmodium, the germ propagated by these conditions and the cause of the affections.

The observation that diseases were communicated to man from the lower animals led to the study of glanders, anthrax, malignant pustule, chicken-cholera and hog-cholera, and, finally, to the discovery that tuberculosis in the lower animals, particularly in cows, through the milk supply, was an exceedingly frequent and dangerous method by which tuberculosis can be communicated, and has led to the present salutary measures adopted by our State Board of Health in exterminating the disease.

The importance of sanitary and antiseptic measures in the military service is illustrated by the fact that in the middle ages, and down to the time of the Crimean War, it was the rule for a greater number of soldiers to die of disease than from wounds. But, in recent years, in countries where sanitary measures have been observed, the relative fatality due to disease has been greatly diminished. The most notable exception to this is our recent Spanish-American War. Much disease in this case may be attributed to the radical change of climate and an imperfect knowledge of the disease peculiar to those regions; but by far the most potent cause was the utter disregard of sanitary measures by the War Department, which completely lost sight of the fact, that the fighting force of the army depended as much on the health of the men, as on their military equipment.

Again, the value of preventive inoculation for the control of disease is illustrated by the effect of vaccination in arresting small-pox, the first disease in which preventive inoculation was employed. In Germany in 1871, out of a population of 50,000,000, 143,000

lives were lost annually by small-pox. As a result of this great mortality, a law was enacted making vaccination compulsory in the first and tenth year of life, so that today the number of deaths has diminished to only 116 a year. During the Franco-German War, the French army, in which vaccination was not compulsory, lost 23,000 soldiers; while the German army, in which vaccination was compulsory, lost but 273 men. It seems absurd that in spite of this positive evidence, anti-vaccination associations can exist and that people can ignore its beneficial effects. In England, at the present time, the medical profession is greatly embarrassed by the enactment of Parliament interposing the "conscience clause," which practically does away with compulsory vaccination.

Not only are the achievements and discoveries of medical science of vital importance in the prevention of disease, but they are equally valuable in the cure of disease. A few years ago the list of supposedly incurable diseases was a very long one, but today the list is rapidly diminishing. Truly may it be said that the opprobrium of one decade is the glory of the next. Such affections as tetanus, hydrophobia, malignant pustule, diphtheria, myxedema, cretinism and allied diseases which were almost invariably fatal, now have a very low death-rate.

Surgery, that handmaid of medicine, has also contributed its share in benefiting the world and in relieving the ills of mankind. Three hundred years ago it was a bold surgeon who would attempt to amputate a limb or perform any important operation. The patient was stretched upon the table, writhing in agony, and the only means of arresting the hemorrhage was by searing the stump with an hot iron, until Ambrose Paré, in 1564, devised the ligature, which, however, required 100 years to become generally adopted. If the patient survived the operation, septicemia and pyemia were the almost inevitable result. Compare this with the painless, bloodless and aseptic methods of today. Operations may now be successfully performed by a tyro in surgery, that a Sir Astley Cooper, a Syme, or a Valentine Mott would have hesitated to undertake. Surgery has also entered the domain of internal medicine, and removes with impunity from those sacred precincts, the cranial, thoracic, abdominal, and pelvic cavities, diseased conditions which before were uniformly fatal. With the assistance of local anesthesia, the treatment of diseases of the eye, ear, nose, throat, and other cavities, has been revolutionised.

It is only by considering these achievements in medicine and surgery, that we can appreciate the results of Pasteur's first studies and investigations into the cause of fermentation, which have led to such grand results; to the discovery that all infectious diseases, so far as their etiology is known, and that all wound infections or septic processes are caused by germs; to a knowledge of the habitat of germs, and of scientific methods of sanitation; to the discoveries of the toxins that prevent the propagation of germs, or render the system immune; and to the fact that the diagnosis of these diseases is no longer a matter of doubt, but can now be accurately determined by the use of the microscope.

In this connection it is safe to predict that it will be but a comparatively short time when we shall be as thoroughly familiar with the germs which are the specific cause of all contagious and infectious diseases and all forms of inflammatory affections, as we now are with those producing diphtheria, phthisis and cholera; and that the toxin or the method of the destruction of each and every one will be equally well known and as readily applied as that of diphtheria at the present time.

Pasteur's discoveries have also produced an epoch in the attitude of medical thought, as well as in medical science, by tending to do away with isms, schools and theories, and all medical philosophy not founded on demonstrable facts.

The beneficial influence of modern medical science upon civilisation is also seen in the lowered death-rate, as shown by the statistics of England (the only data that have been kept with sufficient accuracy to show any definite percentage), where the death-rate has been lowered 50 per cent. during the last century.

If the death-rate of thirty years ago had prevailed in England during the last ten years, her loss would have been greater than it has actually been by 50,000 lives annually.

The influence of medical science is shown not only in lowering the death-rate, but also in the prolongation of life. According to Justinian, in the sixth century the average length of life was thirty years; and in Geneva, in the seventeenth century, it was only twenty years, which was doubtless due to epidemics. Fifty years ago it was estimated to be thirty-eight years in Europe and America, and now it is between forty-five and fifty years.

In considering the question from an economic point of view, the practical American will ask what are the special advantages to be derived from these results? The answer is, that, besides conserving civilisation in various ways, the health and comfort of the habitants are increased and the resources of the nations are correspondingly augmented.

Estimating a person's life to be worth to the community $50 annually, and supposing the average life has increased ten years, then each person has benefited the commonwealth by $500. In this country, with a population of 60,000,000 in round numbers, the annual value of the prolongation of life can be estimated to be about thirty billions of dollars. To this should be added the value of the time saved by the more rapid recovery from operations and disease. Therefore, it is readily to be seen, that the commercial value of any discovery in medicine can be estimated by simply computing the diminished death-rate resulting therefrom.

The value of such medical research, as applied to the prevention of disease in lower animals, is shown by Chamberland, the Minister of Agriculture in France, who computed that the saving to French agriculture, accruing from the use of Pasteur's vaccine in preventing anthrax among sheep and horned cattle, may be estimated at seven million francs annually.

From a purely commercial point of view, the value of sanitary and medical science is incalculable. The old system of quarantine, which formerly presented a barrier to commerce and paralysed the business of the nation, can now easily be supplanted by scientific sanitation and disinfection. It has recently been estimated that the cost of putting Havana in a sanitary condition would be $10,000,000, but that an outbreak of yellow fever in this country would damage the business interests to the extent of a $100,000,000, to say nothing of the loss of life. What investment would surpass this? Many other facts like these could be cited, to show the importance to the nation of adopting the most advanced methods of sanitation, and encouraging research by the liberal endowment of biological and experimental laboratories and scientific libraries.

Just as medical science is valuable from a commercial point of view, so it also increases the industrial forces of society. By the prevention of sickness among the laboring classes and the preservation of general health, all the industries of the country are carried on uninterruptedly, and pauperism and public charity are thus indirectly diminished. Moreover, by this uninterrupted prosperity, resulting from improved sanitary conditions, the investment of foreign capital is thereby attracted, and immigration of the most desirable class encouraged. Every other branch of the government relating to our commercial prosperity has been made a special department of governmental supervision, even to the agricultural department, whereas one of the most important branches of our national existence is entirely overlooked, that relating to our national health.

The intellectual and moral conditions of a nation are also promoted by increasing the general health of its people. This is observed in the public schools, were brightness and clearness of intellect and acuteness of moral preception are the accompaniments of cleanliness and health. Squalor and disease are recognised as the parents of ignorance and crime, and every successful effort in destroying the one is a step toward decreasing the prevalence of the other. "Disease is the penalty of violated law, and medical science is ever striving to adjust the balance between the penalty and the crime."

While the advance in medicine and surgery in the last quarter of a century has been phenomenal, many problems still remain unsolved. With the broadening horizon great difficulties are constantly presenting themselves. With the increase of population, the growing complications of society, and the new conditions of human life, it is reasonable to expect that still greater difficulties will be added. While fields of inquiry will yield a great harvest of problems for the statesman, the sociologist, philosopher and the theologian to solve, the scientist, and especially the physician, will also have his share in the labor and in the reward of this harvest.

Since the sad experience of the crowding in unsanitary cities in the past will not be repeated, it still remains a fact that great cities are the natural fruit of advancing civilisation. No sooner is a country developed than at once a city, almost an empire in extent and diversity of industry, springs up as an emporium of produce and a meeting place for capital and labor.

A quarter of a century ago you could count the millionaires on your fingers. Now what is their number? In the days of our great grandsires, when every man had to get his living directly from the soil, or approximately so, wealth was accumulated only after a generation of thrift and economy; now a man becomes a millionaire in a few years and amasses a fortune by a simple exchange of papers.

How intense is the activity of our times! A man who is not fairly on the run to keep up with the activity and thought of the times will certainly be crushed under the wheels of progress, or miss the tide "which taken at its flood leads on to fortune." Men now live their three score and ten years in a short time, with the apparent satisfaction, however, that "better fifty years of Europe than a cycle of

Cathay." As a result of this intense activity and the stress of industrial competition, we see rising new forms of calamity and distress—the woes of wealth and the pains of pauperism. Luxury and poverty have each diseases peculiar to itself. The inactive has to pay doubly for his leisure, while the overworked pays tenfold for his daily bread.

The difference, from a medical point of view, between these two extremes of civilisation, are widening and increasing in a geometric ratio; and the consequent diseases which will be expected to follow must be met by the alleviating hand of science. Old diseases, we have reason to expect, will assume such different types and degrees of severity as to constitute new diseases. New species of germs may also spring into activity, causing new varieties of infectious diseases. Science, however, will be ever alive to the situation, and ever equal to the new condition. The old formidable enemy to science, in the form of ignorance and prejudice, is passing away, and with the encouragement of national patronage, and the fostering hand of wealth which is beginning to be extended, methods of research will be instituted which will solve all the difficult problems that may arise. As Spencer says: "There is no real progress until scientific knowledge becomes common knowledge and the results of science made the common heritage and privilege of all." The truth of Copernicus was not accepted until proved by Galileo and demonstrated to all mankind; so the discoveries of Pasteur were of little service until their adoption was universal. The eagerness with which the sciences are now being taught in all the colleges and universities, and their principles utilised by the public is not only an evidence of the generous interest awakened in them and a recognition of their importance to civilisation, but an indication that the sciences themselves will derive equal benefits from these investigations, and that in the future still greater and more rapid advancement will be made than during any period of the past.

A new day for medicine is now dawning, when tradition, hypothesis and imperial authority no longer hinder human progress; and speculative and metaphysical methods, that have so long mystified the intellectual world, are now disappearing before the light of more substantial reason and methods based upon the discovery and demonstration of facts and their relation to the laws of nature. This dawning day is the beginning of a new era, when utility will be the aim of all scientific investigation, and the consensus of observation and demonstration, reduced to mathematical precision, will be the criterion of belief and experience, "when the charms of empiricism, and the perplexities of individualism, the enigmas of prognosis, and the dark mystery of those protoplasmic laborers of the economy will vanish before the bright searchlight of the *x*-ray, bacteriology and physiological chemistry."

The fields in which we may expect the greatest discoveries are in bacteriology and physiological chemistry, which together with the other sciences as most valuable allies, the clinician may utilise and adapt all the rich stores of modern knowledge to therapeutic ends, and thus become the promoter of a higher and healthier civilisation.

Medical science, having fought many battles for life and liberty in the dark valleys of the past, now stands a victor on the summit of the nineteenth century, looking forward into the twentieth, and like Hannibal of old, shouts exultingly to brave and daring followers, "Beyond the Alps lies Italy."

